# A comparison between measurement properties of four shoulder-related outcome measures in Nepalese patients with shoulder pain

**DOI:** 10.1007/s11136-022-03080-8

**Published:** 2022-01-24

**Authors:** Sudarshan KC, Saurab Sharma, Karen Ginn, Darren Reed

**Affiliations:** 1 School of Medical Sciences, Faculty of Medicine and Health, University of Sydney, Sydney, NSW 2006 Australia; 2 Centre for Pain IMPACT, Neuroscience Research Australia, Sydney, Australia; 3 Faculty of Medicine and Health, School of Health Sciences, University of New South Wales, Sydney, Australia; 4 Department of Surgical Sciences, Dunedin School of Medicine, Centre for Musculoskeletal Outcomes Research, University of Otago, Dunedin, New Zealand

**Keywords:** DASH/Quick-DASH, SPADI, PSFS, Measurement properties, Shoulder

## Abstract

**Purpose:**

The Patient-Specific Functional Scale (PSFS), Disability of the Arm, Shoulder and Hand (DASH), Quick-DASH, and Shoulder Pain and Disability Index (SPADI) are frequently used instruments in shoulder functional assessment. They are available in Nepali and all but the PSFS has been validated for shoulder assessment. Therefore, the aim of this study was to validate the Nepali PSFS in shoulder pain patients and to compare validity, reliability, and responsiveness of all four instruments to provide a recommendation for their use.

**Method:**

Patients attending physiotherapy completed the Nepali PSFS at baseline and follow-up (1–3 weeks). It was tested for reliability using internal consistency (Cronbach’s *α*), intraclass correlation coefficient (ICC), construct validity by hypothesis testing and responsiveness by anchor-based method using Area Under the Curve (AUC). The instruments were compared based on reported measurement properties and patients’ preference.

**Results:**

156 patients enrolled at baseline and 121 at follow-up. The PSFS showed sufficient reliability (*α* = 0.70, ICC = 0.82), construct validity (all three hypotheses met) and responsiveness (AUC = 0.83). Measurement property comparison demonstrated adequate reliability and validity, while PSFS was the most responsive instrument. Patients favoured the verbal rating scale of the DASH/Quick-DASH. The DASH had a lower completion rate for ‘culturally sensitive’ and ‘uncommon’ activities.

**Conclusion:**

The Nepali PSFS is a reliable, valid, and responsive instrument in shoulder functional assessment. The combined use of the Quick-DASH or SPADI with the PSFS is recommended for a comprehensive assessment of Nepali shoulder pain patients in clinical and research settings. They are shorter, more appropriate to the Nepali context and provide balanced self-evaluation.

## Introduction

Patient-reported outcome measures (PROMs) are increasingly used in the assessment and management of musculoskeletal disorders [1]. They have also become important primary outcome measures in research providing objective scores of patients’ level of self-perceived symptoms and/or disability [1]. Many region-specific PROMs are available, including more than 30 for the shoulder region. These PROMs should be developed according to recommended guidelines and be tested for their measurement properties before they can be endorsed and used clinically and in research [2, 3]. However, with varying degrees of compliance with stated development guidelines and the sheer number of PROMs available, shoulder clinicians can feel confused as to which PROM to use in individual circumstances.

The Disability of Arm, Shoulder, and Hand (DASH) [1], the shorter version of the DASH (Quick-DASH) [4] and the Shoulder Pain and Disability Index (SPADI) [5] are among the most frequently used and recommended shoulder PROMs in both clinical and research fields with the measurement properties of each supported by good quality evidence [3]. Previous studies have suggested that using two PROMs provides a more balanced measure of shoulder function [6, 7]. These three PROMs are available in multiple languages [3, 8] including recently published Nepali versions [9–11]. They are the only available shoulder or upper limb specific tools in Nepali, the national language of Nepal.

Another PROM available in Nepali is the Patient-Specific Functional Scale (PSFS) developed to quantify activity limitations and physical function in any health condition and body part [12, 13]. It is short and the patient chooses three to five of their own relevant activities to use as the assessment items. The English version of the PSFS has been validated in various health conditions including shoulder disorders and has been compared favourably to other musculoskeletal instruments resulting in it being a commonly used instrument in physiotherapy practice [6, 13, 14]. A recent systematic review has endorsed the PSFS as a reliable, valid and responsive PROM for the assessment of physical function in shoulder disorder/pain [15]. The PSFS has been validated for use in Nepali patients with general musculoskeletal pain with predominantly spinal and knee pain [16] but its reliability, validity, and responsiveness properties have not been assessed in a shoulder pain population in Nepal. Additionally, no comparison has been made between the three Nepali shoulder PROMs and the PSFS. A comparison of these four PROMs would provide guidance for Nepali clinicians and researchers as to which PROM is the most suitable for use in patients with shoulder pain.

Therefore, the purposes of this study were to determine the measurement properties of the PSFS in Nepalese patients with shoulder pain and to compare the comprehensibility and measurement properties of the Nepali DASH, Quick-DASH, SPADI and PSFS instruments in order to provide a recommendation for use in clinical practice and research.

## Material and methods

This multicentred longitudinal cohort study was approved by the Institutional Review Committee of Kathmandu University School of Medical Sciences, Nepal (Ref. No. 63/16) and was conducted over a six-month period from June to Dec 2016.

### Patients and procedures

Patients receiving treatment from out-patient physiotherapy departments of three hospitals were screened by a physiotherapist for eligibility and volunteered for this study. These hospitals included a not-for-profit community-based hospital (Dhulikhel Hospital, Dhulikhel), a general urban hospital (Medicare Hospital, Kathmandu) and a large orthopaedic hospital (Nepal Orthopaedic Hospital, Kathmandu). To be included they had to be aged > 18 years, have adequate command of Nepali and have presented to the physiotherapy departments with the primary complaint of shoulder pain. Shoulder pain was defined as pain over the anterolateral, proximal aspect of the shoulder and/or upper arm, which was aggravated by shoulder movements. Patients were also required to test positive to one of the following: Hawkins–Kennedy test, Neer’s impingement test or resisted isometric manual muscle tests (abduction, external/internal rotation). Patients with cervical spine symptoms (pain on neck movements, pain in a dermatomal pattern and/or upper limb paraesthesia), pain of systemic or bioplastic origin were excluded from the study. Prior to the enrolment, all patients provided informed consent. Eligible patients completed the Nepali SPADI, DASH/Quick-DASH and the PSFS (as printed forms) at their first visit and again after an interval of 1–3 weeks. An interview method was used for illiterate patients with no prompting from the assessor. The Nepali version of the Global Rating of Change (GROC-NP) score was also included at the follow-up visit to categorise the patients into stable and improved groups.

### Nepali outcome measures

The Nepali version of the DASH (DASH-NP) [9] is a valid and reliable upper limb assessment tool consisting of 30 items. The Nepali Quick-DASH (QuickDASH-NP) [10] is a subset of the DASH-NP that contains 11 items. Each item on both scales is assessed using a five-point Likert scale ranging from “No symptoms/difficulty” to “Worst symptoms/extreme difficulty”. The Nepali DASH has been reported to measure gross motor function, symptoms, fine motor tasks and recreational activities [9] and the Nepali Quick-DASH symptoms and functions [10]. If more than three items on the DASH-NP and one item on the QuickDASH-NP tools are missed, a valid calculation is not possible. Higher scores indicate higher intensity of the symptoms or disability and calculated using the formula:

$$\begin{gathered} {\text{DASH or Quick}} - {\text{DASH sum score }} = \, \left[ {\left( {{\text{sum of n responses }}/{\text{ n}}} \right) \, - { 1}} \right] \times {\text{25}} \hfill \\ \left( {\text{where n is equal to the number of completed responses}} \right). \hfill \\ \end{gathered}$$

The Nepali version of the SPADI (SPADI-NP) contains 13 items (five pain items and eight disability items) with patients rating their pain and disability symptoms on a numerical rating scale ranging from 0 (No pain/difficulty) to 10 (Extremely painful/difficulty) [11]. The Nepali SPADI assesses symptoms under pain and disability constructs with acceptable measurement properties [11]. More than two items left unanswered results in an invalid score. A higher score indicates greater disability and pain is calculated as

$${\text{SPADI score }} = \, \left( {{\text{total score }}/{13}0} \right){\text{ x 1}}00 \, = \, \%$$

The Nepali version of the PSFS (PSFS-NP) enables patients to identify and self-nominate three to five activities they are unable or have difficulty performing as a result of their health problems. The activities are rated on a scale of 0 (unable to perform) to 10 (Able to perform at prior level) [12]. The scoring is in the reverse order of the SPADI, DASH and Quick-DASH, therefore, higher scores indicate less disability. A minimum of three functional activities is required to produce a valid calculation. PSFS score is calculated using the formula:

$${\text{PSFS score }} = {\text{ sum of activities score }}/{\text{number of activities}}$$

The GROC-NP uses a 7-point scale to measure change in patients’ overall health status. The middle indicator ‘4’ denotes “No change”, > 4 indicate progressive incremental improvements (5—small, 6—moderate, 7—large) and < 4 indicate worsening symptoms [17]. Patients scoring GROC-4 were considered in the ‘stable group’ and GROC-5, 6 and 7 as ‘improved group’.

### Study design

A two-stage study was conducted. The first stage included determination of measurement properties of the PSFS-NP in Nepali patients with shoulder pain and the second stage comparison between the measurement properties of the DASH-NP, QuickDASH-NP, SPADI-NP, and the PSFS-NP.

#### Stage one

##### Measurement properties analysis of the Nepali version of Patient-Specific Functional Scale (PSFS-NP)

The following measurement properties were tested for the PSFS-NP in accordance with the Consensus-based Standards for selection of health status Measurement INstruments (COSMIN) [18] recommendations:

##### Reliability

(i) Internal consistency—evaluated using Cronbach’s alpha (α) and was considered acceptable if α > 0.70 [19].
(ii) Test–retest reliability—confirmed using intraclass correlation coefficient (ICC 2, 1 agreement) with ICC > 0.70 ‘adequate’ in the stable group based on GROC categorisation [20].
(iii) Measurement errors—also called observational error including random and systematic errors, were estimated by the standard error of measurement (SEM) and smallest detectable change (SDC) using the following formulae:
  - SEM = standard deviation (pooled SD) multiplied by (1—ICC)^1/2^ and
  - SDC = z x √2 × SEM where z = 1.96 (z score is estimating a 95% confidence interval).

##### Validity

Construct validity of the PSFS-NP was assessed by hypothesis testing using Pearson’s correlations [21]. Three *a-priori* hypotheses were formulated:

The PSFS-NP would have a moderate to high negative correlation with the

(i) SPADI-NP,
(ii) DASH-NP, and
(iii) QuickDASH-NP.

These correlations would be negative as the PSFS-NP scores in the reverse order to the other three PROMs and moderate to high as previous research would suggest [7, 15]). A correlation < -0.30 was considered weak, − 0.30 to − 0.70 moderate and > -0.70 high [22]. Sufficient construct validity was determined if > 75% of the hypotheses were confirmed (i.e. all three hypotheses).

##### Responsiveness

An anchor-based method, Receiver Operating Characteristics (ROC) curve, using an external criterion (GROC-NP) was used to examine the responsiveness of the PSFS-NP. The ROC curve was plotted for the difference of PSFS-NP scores at baseline and follow-up administration between the stable group (GROC-NP score 4) and the improved groups (GROC-NP scores 5, 6, 7). The Area Under the Curve (AUC) > 0.70 was considered the cut-off value for sufficient responsiveness [18].

Minimal Important Change (MIC) represents interpretability, and it was based on the optimal balance between sensitivity and specificity in the ROC curve.

Data were entered in an Excel spreadsheet [23] and later transferred into SPSS version 24 [24] for statistical analysis.

#### Stage two

##### Participant feedback

Feedback was acquired using a cognitive debriefing interview [25] from the first five participants to assess the comprehensibility of the DASH-NP, QuickDASH-NP, and the SPADI-NP. Probing questions (in Nepali) were used in the interviews and included:

(i) Did you find any difficulty while completing each instrument?
(ii) Were the instructions and items easy to understand?
(iii) Were all items relevant to your shoulder symptoms?
(iv) Was the scoring method used in each instrument easy to answer? If not, why?
(v) Did you leave any item blank? If yes, why?

##### Comparison between measurement properties of four instruments

The measurement properties of the DASH-NP, SPADI-NP and QuickDASH-NP have been reported previously and data extracted from the published manuscripts [9–11]. The measurement properties of the PSFS-NP were obtained from the first stage of this current study.

## Results

### Patients

The first five participants completed all four PROMs and provided feedback on the utility of the instruments. Their data were not included in further measurement property analysis. A further 156 Nepali-speaking patients with shoulder pain (81F:75 M, 47.7 ± 13.5 years) completed the DASH-NP/QuickDASH-NP, SPADI-NP, and PSFS-NP at baseline. Demographic information of the first five participants is described individually in Table 1 and for the additional 156 patients in Table 2. Six patients (4%) missed more than three items of the DASH-NP at the initial and/or follow-up assessment. No patients missed more than one item in the QuickDASH-NP or more than two items in the SPADI-NP, indicating a 100% completion rate. No items were missed in the PSFS-NP.

**Table 1:** Demographic information of initial five participants

Participant	Age	Sex	Education	Occupation	Literacy	Geographical location
#1	35	Male	Yes	Business	L	Village
#2	22	Female	Yes	Student	L	Urban
#3	33	Female	No	Housework	I	Village
#4	45	Male	Yes	Office	L	Urban
#5	> 60	Female	No	No work	I	Urban

*I* Illiterate, *L* literate

**Table 2:** Demographic information of cohort of 156 shoulder pain patients

Items	Mean ± SD	n (%)
Age	47.7 ± 13.5 years	
*Gender*		
Male		75 (48)
Female		81 (52)
*Religion*		
Hindu		105 (67)
Buddhism		36 (23)
Others		15 (10)
*Literacy status*		
No education (illiterate)		70 (45)
Education (literate)		
Primary		58 (37)
Secondary		14 (9)
Bachelor and above		14 (9)
*Occupation*		
Business		27 (17)
Office		17 (11)
Agriculture		11 (7)
Student		6 (4)
Others incl. housework		95 (61)
*Mean scores*	Initial/follow-up (%)	
PSFS	30 ± 22 / 49 ± 25	
SPADI	46 ± 24 / 35 ± 22	
DASH	35 ± 20 / 27 ± 19	
Quick-DASH	34 ± 20 / 25 ± 18	

*DASH* disability of arm, shoulder and hand, *PSFS* patient-specific functional scale, *SD* standard deviation, *SPADI* shoulder pain and disability index (note- higher PSFS scores and lower SPADI/DASH/Quick-DASH scores indicate improved function)

Table 3 provides a summary of the number of patients who missed items in each instrument with the possible reasons given from the cognitive interviews. All four PROMs, including the GROC-NP were completed by 121 patients (78%) at the follow-up visit. Of these, 89 were improved and 32 unchanged according the GROC-NP scores.

**Table 3:** Reasons for omission of items in each instrument

PROMs	Items	*n*	Reason for omission, response of participants (#n)
DASH-NP	Q 17. Recreational activities which require little effort (e.g. Cardplaying, Knitting, etc.)	7	“Not common”—(#2)
	Q 18. Recreational activities in which you take some force or impact through your arm, shoulder, or hand (e.g. Batting in cricket, Volleyball, etc.)	17	“Uncommon activities”—(#3) “I’m too old for these activities”—(#5)
	Q 19. Recreational activities in which you move your arm freely (e.g. Bowling in cricket, Table tennis, Badminton, etc.)	62	“Not commonly involved in such activities”—(#3) “I’m too old for these activities”—(#5)
	Q 21. Sexual activities	61	“Uncomfortable to answer”- culturally sensitive (#1, #3, #4, #5)
QuickDASH-NP	Q 6. Recreational activities in which you take some force or impact through your arm, shoulder, or hand (e.g. Batting in cricket, Volleyball, etc.)	17	“Uncommon activities”– (#3) “I’m too old for these activities”—(#5)
SPADI-NP	Q 10. Putting on your pants/trousers?	2	“Never worn pants/trousers” (#3, #5)

*DASH* disability, arm, shoulder and hands, *SPADI* shoulder pain and disability index, *n* number of patients, *#n* participant number from cognitive interview

### Measurement properties of Nepali version of the Patient-Specific Functional Scale

**Fig. 1:**
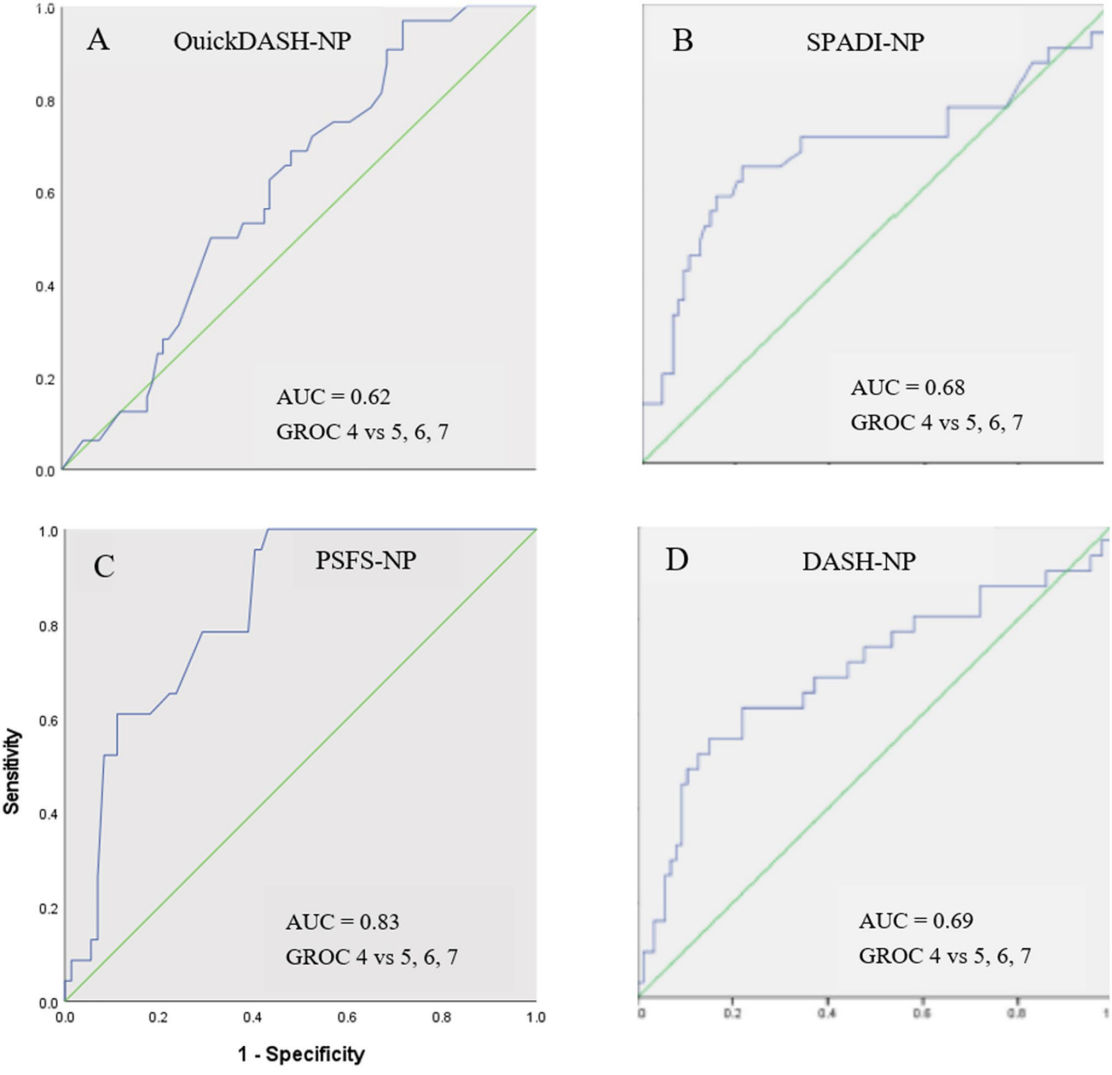
Receivers operating characteristics (ROC) curve between stable and improved patients

Internal consistency (*α* = 0.70) and test–retest reliability (ICC = 0.82, 95% CI 0.60–0.92) of the PSFS-NP were adequate. Standard error of measurement was 0.83 points and SDC 2.30 points of 10. Construct validity of the PSFS-NP was sufficient with all three *a-priori* hypotheses confirmed showing negative and moderate correlations of the PSFS-NP with (i) the SPADI-NP (*r* = − 0.34), (ii) DASH-NP (*r* = -0.32), and (iii) QuickDASH-NP (*r* = -0.34). The result of the ROC curve analysis (Fig. 1) indicates that the PSFS-NP is responsive with a high AUC value of 0.83 (95% CI 0.74–0.91) and MIC 2.66 out of 10 points.

### Participant feedback

The first five participants reported no difficulty in completing the instruments and suggested all the items and instructions were easy to understand. Three items from the DASH-NP and one from the QuickDASH-NP were reported as difficult to answer. Items 18 and 19 (corresponding to item 6 in the QuickDASH-NP) asking about recreational activities were described as not common in the village setting and for the age of the participant and item 21 referring to “sexual activity” was a culturally sensitive item. Item 10 from the SPADI-NP (level of difficulty when putting on trousers/pants) was also not completed by two female participants in the pretesting stating that they wore traditional female garments comprising a long drape described as “Dhotis or Saris” not trousers. The response options used in the instruments were also suggested to be easy to select. No additional suggestions for modification were proposed. However, verbal response scales (DASH-NP and QuickDASH-NP) were favoured by the interviewed participants over numerical response scales (PSFS-NP and SPADI-NP).

### Comparison of the measurement properties of the four instruments

Data for each measurement property for the DASH-NP, QuickDASH-NP, SPADI-NP, and the PSFS-NP are summarised in Table 4. All four instruments demonstrated adequate reliability and validity. The responsiveness was acceptable for the DASH-NP and the SPADI-NP, weak for the QuickDASH-NP and excellent for the PSFS-NP. The SDC values for all four instruments were lower than the MIC values indicating all four instruments can detect clinically meaningful change over time in Nepalese patients with shoulder pain.

**Table 4:** Comparison between SPADI-NP, DASH-NP, QuickDASH-NP, and PSFS-NP

Measurement properties	SPADI-NP [11]	DASH-NP [9]	QuickDASH-NP [10]	PSFS-NP
*Reliability*				
Internal consistency (α)	0.82 (P), 0.88 (D) 0.90 (T)	0.92	0.79 (F), 0.75 (D) 0.82 (T)	0.70
Test–retest reliability (ICC) (95% CI)	(P) 0.89 (0.80–0.95) (D) 0.96 (0.92–0.98) (T) 0.95(0.90–0.97)	0.97 (0.94–0.98)	(F) 0.91(0.82–0.96) (D) 0.97(0.94–0.99) (T) 0.94 (0.92–0.98)	0.82 (0.60–0.92)
Standard error of measurement Smallest detectable change	2.1/100 5.7/100	3.97/100 11/100	2.83/100 7.84/100	0.83/10 2.30/10
*Validity (correlation = r)*				
SPADI DASH Quick-DASH PSFS	1 0.63(P), 0.81(D) 0.81 − 0.34	0.63(P), 0.81(D) 1 0.96 − 0.32	0.81 0.96 1 − 0.33	− 0.34 − 0.32 − 0.33 1
*Responsiveness*				
AUC (95% CI)	0.68 (0.55–0.81)	0.69 (0.57–0.81)	0.62 (0.50–0.73)	0.83 (0.74–0.91)
MIC	12.3/100	11.2/100	11.02/100	2.66/10

*AUC* area under the curve, *CI* confidence interval, *D* disability, *DASH* disability of arm, Shoulder and Hand, *F* function, *ICC* intraclass coefficient correlation, *MIC* minimal important change, *P* pain, *PSFS* patient-specific functional scale, *SPADI* shoulder pain and disability index, *T* total

## Discussion

### Measurement properties of the Nepali version of the Patient specific functional scale

The Nepali version of the PSFS demonstrated sufficient reliability, validity, and responsiveness in Nepalese patients with shoulder pain. The reliability and validity of the PSFS-NP in shoulder pain were comparable to the original English version (ICC = 0.70, *r* = 0.51 & 0.59) [26] and other translated versions in shoulder disorders (ICC = 0.83 & 0.87, *r* = 0.45 & 0.55) [7, 13]. The results are also consistent with testing of the PSFS-NP in other areas of musculoskeletal pain (ICC = 0.75, *r* = 0.32 & 0.47) [16] providing strong evidence for the versatility of the PSFS-NP in a Nepali context. The result of the responsiveness testing was sufficient with values of AUC (0.83) similar or higher than previously reported (AUC = 0.67, 0.75, & 0.83) [13, 26, 27] and suggests the PSFS-NP is a good option to assess change in physical function in Nepalese adults with shoulder pain.

### Participant feedback on comprehensibility

While participant feedback suggested there were no issues of understanding with the instructions, items and the response scales used in the three shoulder-specific PROMs (DASH-NP, QuickDASH-NP, SPADI-NP) investigated, some items were flagged as difficult to answer for cultural reasons, particularly in the DASH-NP (items 19 and 21) and the QuickDASH-NP (item 6). Item 19 from the DASH-NP (corresponding item in the QuickDASH-NP—item 6) was indicated as an uncommon recreational activity by two participants (#3, 5). Similarly, the item referring to sexual activity in the DASH-NP (item 21) was identified as culturally sensitive with four out of five participants (#1, 3, 4, 5). This result was not unexpected as these items have been highlighted in previous translations as questions that were culturally sensitive [28, 29]. Item 10 (relating to wearing pants or trousers) from the SPADI-NP was left by two women included in the debriefing interview (#3 and 5, aged 35 and > 60) stating, “I have never worn pants/trousers because I am a female”.

Although, some items were not answered, 100% (*n* = 156) of the QuickDASH-NP and SPADI-NP instruments were valid (i.e. no more than one question left blank) and a lower, but still acceptable, 96% (*n* = 150) of the DASH-NP instrument (no more than three questions left blank) [10]. The items of the DASH-NP which were left unanswered by the largest number of participants related to sexual activity and recreational activities and were not included in the QuickDASH-NP. Despite these items having previously been flagged as problematic the DASH has been used widely as a valid and reliable multi-factor tool to assess shoulder function in multiple languages and shoulder conditions [28, 29]. An advantage of the PSFS-NP is that all items are self-selected and consequently no person logically would choose an activity they did not feel comfortable reporting or in which they did not engage. In Nepal, with its culturally sensitive population, the QuickDASH-NP, SPADI-NP and the PSFS-NP may be more appropriate, with less chance of items being left blank.

The verbal rating scale (five-point Likert scale) of the DASH-NP and QuickDASH-NP was favoured over the numerical rating scale (NRS) (score 0–10). Higher error rates using the NRS over the verbal scale have been reported in low-socioeconomic countries such as Nepal, with advanced age and lower education levels proposed as the likely reason [30]. This finding was also reported previously for the Nepali PSFS in Nepalese patients with musculoskeletal pain. For this reason the PSFS-NP was trialled with a 7-point verbal rating scale however, it did not improve error rates [31]. This evidence suggests both the verbal and numerical responses are comparable in the Nepali context, but a preference may be for the verbal scale.

The shorter instruments, the SPADI-NP, QuickDASH-NP, and PSFS-NP have a distinct advantage over the full DASH-NP with considerably less time needed to complete the form. This has been estimated at less than two minutes [6]. The shorter instruments may be more practical with decreased administrative burden to Nepali patients, clinicians, and researchers in explaining the instruments and gathering results. However, the short nature of the PSFS-NP may also be a disadvantage. With only three to five patient-nominated activities selected, it may not adequately capture a broad range of functional activities and limit its comprehensibility.

### Measurement properties of the four instruments

The DASH-NP, QuickDASH-NP and the SPADI-NP have all demonstrated sufficient reliability and validity while responsiveness testing suggests that PSFS-NP is the most responsive with a higher AUC value (0.83) than the other three shoulder PROMs. Previous studies have reported lower responsiveness for the PSFS-NP than other shoulder-related instruments [13, 27]. A suggested explanation is that the PSFS-NP is both patient and function specific. Only the single construct of disability is measured and therefore, the GROC-NP may have captured perceived improvement only based on level of disability of the chosen specific activity rather than overall change in shoulder symptoms [27]. The DASH-NP, QuickDASH-NP, and SPADI-NP instruments (multi-factors) require patients to record not only disability symptoms but also pain and/or other aspects such as impact on sleeping and mental health symptoms which are more inclusive of the whole biopsychosocial pain response. The MIC values for all four scales were higher than the SDC values of their respective instruments indicating that these four scales are suitable to obtain patient-perceived change, which is both statistically significant and important.

The use of any of these PROMs would be acceptable and it may come down to the preference of the patients, clinicians, or researchers. However, considering other factors such as time required to fill in the forms, compliance with items that are culturally sensitive and content diversity of the PROMs, the concurrent use of either the QuickDASH-NP and the PSFS-NP or the SPADI-NP and the PSFS-NP would be recommended for use in Nepalese shoulder pain patients in both clinical and research settings. This is consistent with the recommendations for English shoulder PROMs which suggest a more balanced and accurate assessment is provided with a combination of two short PROMs (the SPADI and the QuickDASH) [6].

### Strength and limitation

Data comparing shoulder PROMs in non-English languages are limited and as far as we are aware, this is the first study to make a comparison of the content validity (comprehensibility) of shoulder PROMs in non-English languages. This study followed the COSMIN guidelines for measurement property testing with a large cohort representing a broad range of the population between 18 to 65 years, with equal male and females and importantly included literate/illiterate patients from urban/regional areas, increasing its heterogeneity. While qualitative evidence is reported in the current study, it was not designed as qualitative research and therefore, a more thorough qualitative analysis and reporting was not possible. However, an interview using probing questions with a small number of patients such as in this study is considered adequate by COSMIN guidelines [25] to assess comprehensibility and therefore, relevant feedback from the patients is eminently valuable in comparing instruments. The short time frame recommended and used in this study for follow-up (1–3 weeks) was designed to limit an overall improvement in patients shoulder symptoms and prevent recall bias, but further measurement testing using a longer time frame may be beneficial to confirm the responsiveness of these instruments. Further testing where the DASH-NP and QuickDASH-NP are independently and concurrently administered may also provide a different comparison than presented in this current study [10]. A larger sample size for the estimation of the reliability, responsiveness and MIC may have provided a greater clarity to test–retest reliability, responsiveness and interpretability (MIC). Estimation of floor and ceiling values, subgrouping of the patients with high and low baseline scores for the MIC purpose and use of predictive modelling approach for MIC estimation may also have given clearer interpretability and a more precise estimation of the MIC.

## Conclusion

There is strong measurement testing evidence to support the use of all four available Nepali instruments (PSFS-NP, SPADI-NP, DASH-NP, QuickDASH-NP) for shoulder pain patients in Nepal and there is no need to continue developing new PROMs or translating other shoulder-related PROMs into Nepali. The combined use of either the QuickDASH-NP or the SPADI-NP with the PSFS-NP would provide a comprehensive self-perceived assessment of Nepalese shoulder pain patients’ symptoms and be recommended for use in research settings and clinical management of shoulder pain in Nepal.

## Data Availability

The data used to provide evidence for this study may be obtained from the authors upon reasonable request and with permission from the research institutions.
